# Social inequalities in leisure-time and transport-related physical activity through the lens of intersectionality: 10-year longitudinal study in Brazil

**DOI:** 10.1186/s12966-026-01900-5

**Published:** 2026-05-05

**Authors:** Andreia Alexandra Machado Miranda, Danilo Dias Santana, Andiara Schwingel, Grace M. Turner, Kiya L. Hurley, Katie L. Edwards, Shelby Keye, Pedro Curi Hallal, Alex Antonio Florindo

**Affiliations:** 1 School of Arts, Sciences and Humanities, University of São Paulo, São Paulo, SP Brazil; 2 Department of Health and Kinesiology, University of Illinois Urbana-Champaign, Champaign, IL USA; 3 School of Sport, Exercise and Rehabilitation Science, University of Birmingham, Birmingham, UK United Kingdom; 4 School of Health Sciences, University of Birmingham, Birmingham, UK United Kingdom; 5 School of Psychology, University of Birmingham, Birmingham, UK United Kingdom

**Keywords:** Social inequalities, Physical activity, Intersectionality, Jeopardy index, Longitudinal study

## Abstract

**Background:**

Social factors shape health behaviors and contribute to persistent inequalities, especially in the Global South. However, few studies have examined how intersecting social identities influence physical activity in low- and middle-income countries. This study explored social inequalities in leisure-time (LTPA) and transport physical activity (TRPA) through the lens of intersectionality among individuals living in São Paulo, Brazil.

**Methods:**

Longitudinal data from 978 participants of the *Health Survey of São Paulo: Physical Activity and Environment* cohort were analyzed from three waves: 2014/2015, 2020/2021, and 2023/2024. Physical activity was measured using the long-form International Physical Activity Questionnaire (IPAQ). To capture intersectional social disadvantage, a Multiple Jeopardy Index was constructed by combining sex, race/skin color, and educational level, with scores ranging from 0 (lower vulnerability) to 4 (higher vulnerability). Associations between Jeopardy Index and physical activity in both domains were assessed using multilevel Poisson regression models, considering repeated measures nested within three hierarchical levels: observations, individuals, and census tracts.

**Results:**

Individuals in the highest vulnerability groups (female, belonging to racial or ethnic minority groups, and with low educational attainment) consistently reported lower levels of LTPA over time. In fully adjusted models, participants in higher vulnerability Jeopardy categories 3 and 4 showed significantly lower prevalence of LTPA compared to the lower vulnerability reference group (category 0: male, White, and highly educated), with prevalence ratios of 0.72 (95% CI: 0.57–0.91) and 0.62 (95% CI: 0.49–0.80), respectively. A clear inverse gradient and significant dose-response trend was observed (*p* < 0.001). Regarding TRPA, prevalence was higher among males and younger participants, and also more frequent among individuals from racialized or minority groups and those with a high school education. However, no significant associations were found between the Jeopardy Index and TRPA in any model.

**Conclusions:**

Persistent and widening inequalities in LTPA were observed among Brazilians over a 10-year period, with intersections of sex, race/skin color, and education disproportionately affecting the most vulnerable groups, particularly women from racialized or minority groups with lower educational attainment. Public policies and programs should prioritize socially disadvantaged groups by promoting inclusive and sustained opportunities for leisure-time activity.

**Supplementary Information:**

The online version contains supplementary material available at 10.1186/s12966-026-01900-5.

## Background

Physical activity is widely recognized as a key factor in promoting health, preventing non-communicable diseases [1–3], and improving mental health by reducing the risk of depression and anxiety, while enhancing psychological well-being and quality of life [4, 5].

In public health research, physical activity is usually examined by domain, with leisure-time (e.g., sport, exercise) and transportation (e.g., walking or cycling for transport) being the most relevant in the Brazilian context. Despite their importance, overall physical activity levels in Brazil remain low: only 34.2% of men and 26.4% of women aged 18 years or older meet the recommended levels of leisure-time physical activity (LTPA), and 31.7% engage in transport-related physical activity (TRPA), mostly walking [6].

Although both LTPA and TRPA provide health benefits, their impacts may differ. LTPA is typically undertaken by choice and with intention, often in structured or supportive settings, and is associated with greater physical and mental health gains [4, 5]. TRPA also contributes to physical health but may not confer the same psychological or mental health benefits, especially when undertaken out of necessity in unsafe or stressful environments, conditions more prevalent among disadvantaged populations [7–9].

Such environmental and social disadvantages are particularly evident in metropolitan areas of Brazil, such as São Paulo, where socio-spatial segregation intensifies disparities in access to safe environments for physical activity. Residents of peripheral neighborhoods are more likely to face exposure to violence, inadequate lighting, lack of public leisure spaces, and poor transport infrastructure. Urban planning and infrastructure are highly unequal and generally more deficient in low-income areas and municipalities [10–12]. Evidence from other countries in the Global South, such as Colombia, South Africa, Mexico, and India, reveals similar patterns of social and spatial disadvantage. In these settings, structural and intersectional barriers - linked to income, gender, and race or ethnicity - limit individuals’ ability to engage in healthy behaviors [13–18]. Together, these global patterns underscore the importance of examining physical activity through an equity-oriented lens, particularly in the Global South where structural barriers are often more pronounced.

Social inequalities in physical activity are not shaped by single dimensions of disadvantage, but rather by the accumulation and interaction of multiple social positions [19, 20]. Within this perspective, the Multiple Jeopardy framework provides a useful approach to capture how overlapping social disadvantages compound vulnerability [21–24]. Importantly, the dimensions included in a Jeopardy Index are not fixed or universal; rather, they should be selected based on theoretical relevance, empirical evidence, and contextual specificity. In the present study, sex, race/skin color, and educational attainment were selected because they represent structurally rooted axes of social stratification in Brazil and are consistently associated with inequalities in physical activity. Evidence shows that women are less likely to engage in leisure-time physical activity than men, partly due to gender norms, unequal distribution of unpaid domestic work, caregiving responsibilities, and safety concerns, particularly in urban environments marked by violence and poor infrastructure [11, 13–18]. Racial and ethnic inequalities in physical activity are also well documented, with non-white populations disproportionately exposed to structural barriers such as residential segregation, discrimination, and reduced access to safe and supportive environments for physical activity [12, 16, 17]. Educational attainment, widely used as an indicator of long-term socioeconomic position, is strongly associated with health behaviors, reflecting differential access to resources, information, and opportunities that facilitate engagement in physical activity [18, 25, 26]. These dimensions of disadvantage frequently co-occur and accumulate across the life course, particularly in highly unequal urban settings, providing a strong rationale for their joint consideration within a Multiple Jeopardy framework [22–24].

Despite growing recognition of the social determinants of physical activity, important research gaps persist. Most existing studies rely on cross-sectional designs, limiting our understanding of how intersecting social disadvantages shape different domains of physical activity over time. More integrated analytical approaches are therefore needed to capture the dynamic and cumulative nature of social inequalities in leisure-time and TRPA. Yet, few studies have applied an intersectional lens to examine domain-specific patterns of physical activity longitudinally, particularly in the context of the Global South. Longitudinal assessments are essential to inform equity-oriented strategies and guide the design and evaluation of effective policies aligned with the Global Action Plan [27].

Hence, this study addresses this gap by applying an intersectional approach to longitudinal data to investigate the social determinants of leisure-time physical activity (LTPA) and transport-related physical activity (TRPA) in individuals living in São Paulo, Brazil.

## Methods

### Study design and setting

This study uses data from the Health Survey of São Paulo: Physical Activity and Environment (ISA study), a longitudinal study designed to examine how characteristics of the built environment influence health-related behaviors and outcomes. The study monitors changes in LTPA and TRPA over a 10-year period (3 waves: 2014/2015, 2020/2021, and 2023/2024), and examines how urban infrastructure is associated with sedentary behavior, nutritional status, mobility, mental health, and chronic disease incidence. The ISA study includes individuals living in São Paulo city, Brazil’s largest metropolis and a major economic and urban center in the Global South. As of 2022, São Paulo had an estimated population of 11.45 million, a population density of 7,527.76 inhabitants per km², and a total area of 1,521.2 km² [28].

### Sampling and data collection protocol

The complete study protocol can be found in Florindo et al. [29]. In brief, the baseline data (wave 1) were collected between September 2014 and December 2015. The sample was stratified into five health administrative areas of São Paulo (north, mid-west, southeast, south, and east) and a two-stage cluster sampling design was used. In the first stage, 30 census tracts - the smallest geographical unit for census purposes in Brazil - were selected from each health administrative area, totaling 150 tracts across the city. In the second stage, 5,940 households were randomly selected (an average of 39.6 per census tract). In the final stage, face-to-face interviews were conducted with 4,042 study participants.

Data for the second wave were collected between October 2020 and February 2021. Due to the COVID-19 pandemic, interviews were conducted remotely, over the telephone. This wave included a sample of 1,434 individuals, representing 35.4% of the total baseline sample. In the third wave (February 2023 to September 2024), data were collected through both in-person and telephone interviews. Prior to data collection, a home visit was conducted to update participant information. Computer-assisted telephone interviewing was used for phone interviews, while household interviews were conducted using electronic questionnaires on mobile devices. A total of 1,583 participants were successfully followed up during this wave, corresponding to 39% of the original baseline sample.

For the present analysis, the final study sample included 978 participants who completed all three waves of the study. All interviews were recorded for quality control purposes, and fieldwork was carried out by trained interviewers.

### Physical activity

Self-reported physical activity was assessed using the long version of the International Physical Activity Questionnaire (IPAQ). This is a standardized instrument used to assess the frequency and duration of physical activities in different domains, including leisure-time and transportation. The IPAQ has been translated and validated for use in the Brazilian adult population and is widely used in epidemiological studies across Latin America [30, 31].

LTPA includes exercise, sports, recreational activities, and walking performed during free time. Six questions address walking, moderate, and vigorous activities. Regarding the transport domain (TRPA), participants were asked about walking and cycling as modes of transportation to and from work, school, or other routine destinations. For this study, only walking for transport was analyzed. Cycling was reported by a very small proportion of participants (less than 5.5% across all waves) which limited its analytical power and could compromise the robustness of the estimates if included. In both LTPA and TRPA domains, frequency (days/week) and duration (minutes/day) were assessed using the IPAQ. Weekly minutes were calculated, and values below 10 min per week were recoded as zero, following IPAQ data processing recommendations. For the purposes of this analysis, participation in each domain was subsequently dichotomized as ‘yes’ (≥ 10 minutes/week) or ‘no’ (< 10 minutes/week). This approach was adopted to capture engagement in each domain rather than volume of activity and is commonly used in epidemiological studies to distinguish meaningful participation from non-participation.

### Sociodemographic and other covariates

Sociodemographic and lifestyle information was collected in all waves of the ISA study through standardized and structured questionnaires (see Florindo et al. [29] for more details). To measure intersectionality, the Jeopardy Index was constructed based on the variables sex (male or female), race/skin color (White or Others), and educational level (incomplete elementary school or less, elementary to complete high school or college degree). Educational attainment was used as a proxy for socioeconomic status, reflecting long-term social and economic positioning, and is widely used in epidemiological studies of physical activity [25, 26]. Race/skin color was self-reported during the interviews, and participants were classified as White, Black, Brown (“pardo”), Asian, Indigenous, or Other, according to the categories defined by the Brazilian Institute of Geography and Statistics (IBGE). The “Other” category in the questionnaire included individuals who did not identify with standard classifications and instead used subjective or regional terms, such as “moreno” (typically referring to brown-skinned or dark-haired individuals), “mestiço” (mixed-race), or colloquial expressions like “café com leite” (“coffee with milk”). For the Jeopardy Index, all participants identifying with racial or ethnic minority groups (including Black, Brown, Asian, Indigenous, and IBGE’s Other) were grouped together as “Others”.

Additional covariates included in the multilevel models were age (categorized as 12–29, 30–59, and 60 years or older), marital status (living with a partner - married or in a stable union - or without a partner - single, widowed, or divorced), employment status (employed or unemployed, including retirees, homemakers, and students), and sedentary behavior (daily hours of TV watching). Body mass index (BMI) was calculated as weight in kilograms divided by height in meters squared (kg/m²), and participants were classified into two BMI categories: without overweight (BMI < 25 kg/m² for adults; <27 kg/m² for older adults) and overweight (BMI ≥ 25 kg/m² for adults; ≥27 kg/m² for older adults), according to PAHO/WHO recommendations [32, 33].

### Multiple jeopardy index

This study applied the concept of “multiple jeopardy”, rooted in intersectionality theory [20, 21], to examine how overlapping social disadvantages can compound and intensify inequality. According to this principle, social identities do not operate in isolation; rather, they interact in ways that may amplify individuals’ exposure to social disadvantage and structural vulnerability. To quantify this concept, a composite score was developed which reflects cumulative disadvantage across three dimensions: sex, race/skin color, and educational attainment, with the latter used as a proxy for socioeconomic status.

Each variable was coded to capture increasing levels of social vulnerability. Specifically, scores were assigned to each variable as follows: for sex, males were coded as 0 and females as 1; for race/skin color, individuals identifying as White were coded as 0, while those identifying as Black, Brown, Asian, Indigenous, or Other were coded as 1; for educational attainment, individuals with a college degree were coded as 0, those with complete elementary to complete high school education as 1, and those with incomplete elementary education or less as 2 (Table 1). The points from each dimension were summed to create a Jeopardy Index, with scores ranging from 0 (lower vulnerability) to 4 (higher vulnerability). A score of zero indicated individuals who were male, White, and held a university degree. A score of four was assigned to women who identified with racial or ethnic minority groups and had the lowest educational level (incomplete elementary school or less).

**Table 1:** Scoring system and weights used to construct the Jeopardy Index

Variable	Category	Score (weight)
Sex	Male	0
	Female	1
Race/skin color	White	0
	Others (Black, Brown, Asian, Indigenous, and Other)	1
Educational level	Graduate or postgraduate	0
	Elementary to complete high school	1
	Incomplete elementary school or less	2

#### Statistical analysis

Descriptive analyses were conducted to examine the distribution of sociodemographic characteristics, including sex, age group, race/skin color, and educational level, across the three waves of the ISA study. Chi-square tests were used to compare the frequencies of these characteristics between waves and to assess their associations with the two main outcomes: LTPA and TRPA, at each of the three time points (2014/15, 2020/21, and 2023/24). Additionally, chi-square tests for trend were applied to evaluate changes in the distribution of population characteristics over time and to assess trends in the prevalence of LTPA and TRPA throughout the study period. These tests were also used to analyze trends in LTPA and TRPA within each sociodemographic category over time. This approach enabled us to examine whether differences in physical activity persisted or changed during the study period, particularly among more socially vulnerable groups.

Social inequalities in the two physical activity domains were examined using an intersectional approach designed to capture overlapping dimensions of social disadvantage. To quantify this, the Multiple Jeopardy Index [22] was applied to assess the cumulative burden of disadvantage based on sex, race/skin color, and educational attainment. The prevalence of LTPA and TRPA was calculated for each level of the Jeopardy Index across all three study waves, enabling the evaluation of how compounded social vulnerabilities influenced physical activity patterns over time.

To investigate the association between the Jeopardy Index and the practice of LTPA and TRPA, it was used multilevel mixed-effects Poisson regression models with robust variance estimation to account for the hierarchical data structure and to directly estimate prevalence ratios. The models incorporated three hierarchical levels: repeated observations at each study wave (level 1; *n* = 2,668 person-wave observations), individuals (level 2; *n* = 978), and census tracts (level 3; *n* = 32). The outcomes were binary indicators of engaging in LTPA or TRPA (yes/no), and the main exposure was the Jeopardy Index, categorized into five levels (0 to 4). Analyses were conducted in three stages: (1) crude models; (2) models adjusted for wave of data collection (baseline, 2nd, and 3rd waves); and (3) models additionally adjusted for age group, marital status, employment status, sedentary behavior, and body mass index (BMI).

Covariates were selected a priori based on previous literature and conceptual considerations, with guidance from a directed acyclic graph (DAG) illustrating the hypothesized causal structure linking cumulative social vulnerability (Jeopardy Index), potential confounders, and physical activity outcomes (Supplementary Figure S1).

To ensure that these covariates were independent and did not introduce collinearity in the models, pairwise associations (Cramér’s V) and Variance Inflation Factors (VIFs) were examined in a linear model including all covariates. All VIF values were below 5 (mean VIF = 1.43), indicating no collinearity concerns. Detailed results are provided in the Supplementary Material (Supplementary Table S1).

All analyses were conducted using STATA^®^ statistical software, version 16.1 (Stata Corporation, College Station, TX, USA), and a 5% significance level was adopted for all statistical tests.

## Results

A total of 978 participants with complete data across the three waves of the ISA study were included in the analyses. The main descriptive characteristics of the study population over time are presented in Table 2. The sample comprises 58% females, and 50.4% of individuals self-identified as White, with the 30–59 years age group being the most frequently represented. For educational attainment, there was an increase in the number of participants with college or postgraduate degrees (from 22.4% at baseline to 32.9% in wave 3).

**Table 2:** Characteristics of participants in the ISA study across the 3 waves (N= 978)

	**Frequency (CI 95%)**			*** p for trend***
	**Baseline**	**Wave 2**	**Wave 3**	
Sex				
Male	42.0 (39.0–45.1)			
Female	58.0 (54.8–61.0)			
Age (years)				
12–29	27.0 (24.3–29.9)	21.4 (18.9–24.0)	18.6 (16.3–21.2)	
30–59	47.7 (44.6–50.9)	44.1 (41.0–47.2)	42.1 (39.1–45.2)	**<0.001**
60+	25.2 (22.6–28.1)	34.6 (31.6–37.6)	39.3 (36.2–42.4)	
Race/skin color				
White	50.4 (47.2–53.6)			
Asian	2.5 (1.6–3.7)			
Black	11.5 (9.6–13.7)			
Brown	32.1 (29.2–35.2)			
Others	3.5 (2.3–4.7)			
Educational level				
Incomplete elementary school or less	50.9 (47.8–54.0)	35.7 (32.8–38.8)	35.8 (32.8–38.8)	
Elementary to complete high school	26.7 (24.0–29.5)	35.7 (32.8–38.8)	31.3 (28.4–34.3)	**<0.001**
College degree	22.4 (19.9–25.1)	28.6 (25.8–31.5)	32.9 (30.0–35.9)	
Leisure-time physical activity (LTPA)	38.0 (35.0–41.1)	50.4 (47.3–53.5)	51.4 (48.2–54.5)	**<0.001**
Transport-related physical activity (TRPA)	61.8 (58.7–64.8)	73.2 (70.3–75.9)	71.6 (68.7–74.4)	**<0.001**

*p *value = x^2^ test for linear trend for population outcomes over time

Frequencies are based on participants who took part in all three waves (N = 978), with variable-specific missing data

Bold values indicate statistical significance (p < 0.05).

**Fig. 1:**
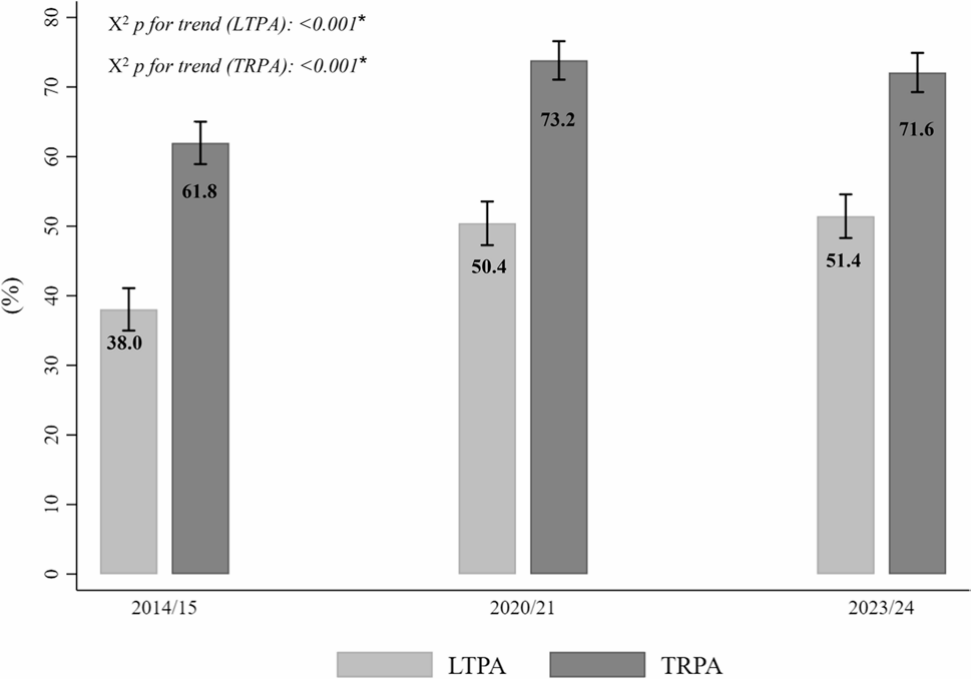
Frequency (%) of leisure-time physical activity (LTPA) and transport-related physical activity (TRPA) among the three waves of the ISA-study *Chi-square tests for linear trend for LTPA and TRPA frequency by time

Variations in the prevalence of LTPA and TRPA across the three time points are shown in Fig. 1. A significant upward trend was observed in both domains, with LTPA increasing from 38.0% at baseline to 51.4% in wave 3 (*p* < 0.001), and walking for transport (TRPA) from 61.8% to 71.6% in the same period (*p* < 0.001).

Overall, LTPA prevalence was highest among males, younger and White individuals, and participants with the highest educational attainment (college degree or higher) (Table 3). For TRPA, prevalence was also higher among males and younger participants; however, it was more frequent among participants identifying with racial or ethnic minority groups and among individuals with a high school education level (Table 3).

**Table 3:** Social characteristics of participants by leisure-time physical activity (LTPA) and transport-related physical activity (TRPA) across the three waves of the ISA study (*N* = 978)

Variables	LTPA (min/week)			*p* for trend	TRPA (min/week)			*p* for trend
	Frequency (CI 95%)				Frequency (CI 95%)			
	Baseline	Wave 2	Wave 3		Baseline	Wave 2	Wave 3	
Sex								
Male	42.6 (37.9–47.4)	58.6 (53.8–63.3)	56.9 (52.0–61.6.0.6)	**< 0.001**	62.3 (57.5–66.8)	77.4 (73.1–81.2)	72.5 (68.0–76.6.0.6)	**< 0.001**
Female	34.7 (30.9–38.8)	44.4 (40.4–48.6)	47.4 (43.4–51.7)	**< 0.001**	61.7 (57.6–65.6)	71.2 (67.4–74.8)	71.8 (67.9–75.3)	**< 0.001**
*p* value	**0.013**	< 0.001	0.003		0.859	**0.032**	0.803	
Age (years)								
12–29	51.1 (45.1–57.1)	60.8 (54.0–67.2.0.2)	61.5 (54.2–68.3)	**0.021**	64.8 (56.6–72.2)	80.9 (74.9–85.6)	76.4 (69.6–82.0)	**0.029**
30–59	33.2 (29.1–37.6)	48.7 (44.0–53.4.0.4)	50.7 (45.9–55.5)	**< 0.001**	63.6 (59.1–67.8)	76.3 (72.1–80.1)	71.8 (67.3–76.0)	**0.005**
60+	33.2 (27.6–39.3)	46.1 (40.9–51.5)	47.4 (42.4–52.4)	**0.001**	55.9 (49.6–61.9)	66.3 (61.1–71.1)	70.3 (65.5–74.7)	**< 0.001**
*p* value	**< 0.001**	0.003	0.007		0.090	< 0.001	0.321	
Race/skin color								
White	40.2 (35.8–44.7)	48.6 (44.1–53.1)	53.7 (49.2–58.2)	**< 0.001**	62.5 (58.1–66.8)	73.6 (69.4–77.4)	68.8 (64.4–72.8)	**0.032**
Others	36.6 (32.3–41.0)	53.1 (48.6–57.6)	50.2 (45.7–54.8)	**< 0.001**	61.6 (57.0–65.9.0.9)	74.7 (70.4–78.5)	75.0 (70.8–78.8)	**< 0.001**
*p* value	0.256	0.169	0.286		0.753	0.714	**0.036**	
Educational level								
Incomplete elementary school or less	34.9 (30.9; 39.2)	44.1 (39.0; 49.4)	41.1 (36.1; 46.4)	**0.044**	59.6 (55.3; 63.9)	70.2 (65.2; 74.8)	70.8 (65.9; 75.4)	**< 0.001**
Elementary to complete high school	37.2 (31.5; 43.2)	54.4 (49.2;59.6)	54.6 (49.0; 60.1)	**< 0.001**	65.5 (59.5; 71.0)	79.1 (74.5; 83.0)	74.5 (69.3; 79.1)	**0.023**
College degree	46.1 (39.6; 52.7)	53.0 (47.2; 58.8)	59.6 (54.2; 64.8)	**0.002**	63.0 (56.4; 69.1)	71.7 (66.1; 76.7)	71.1 (65.9; 75.8)	0.063
*p* value	**0.001**	**0.029**	**< 0.001**		0.054	**0.014**	0.691	

*p* value = x^2^ test for linear trend for LTPA and TRPA within each category of variables over time

*Bold values indicate statistical significance (p < 0.05).*

Table 4 shows the distribution of Jeopardy Index scores over time. The proportion of participants in the lower vulnerability group (score 0 – male, White, with higher education) increased from 6.3% at baseline to 8.6% in Wave 3. In contrast, the proportion in the higher vulnerability group (score 4 – female, belonging to racial or ethnic minority groups, with low education) decreased from 13.3% to 9.9%.

**Table 4:** Distribution of Jeopardy Index scores across the three waves of the ISA study

Jeopardy Index score (*N* = 938)	Frequency *n* (%)		
	Baseline	Wave 2	Wave 3
0 (lower vulnerability group)	59 (6.3)	72 (7.7)	81 (8.6)
1	189 (20.2)	222 (23.7)	221 (23.6)
2	260 (27.7)	271 (28.9)	278 (29.6)
3	305 (32.5)	278 (29.6)	265 (28.3)
4 (higher vulnerability group)	125 (13.3)	95 (10.1)	93 (9.9)

**Fig. 2:**
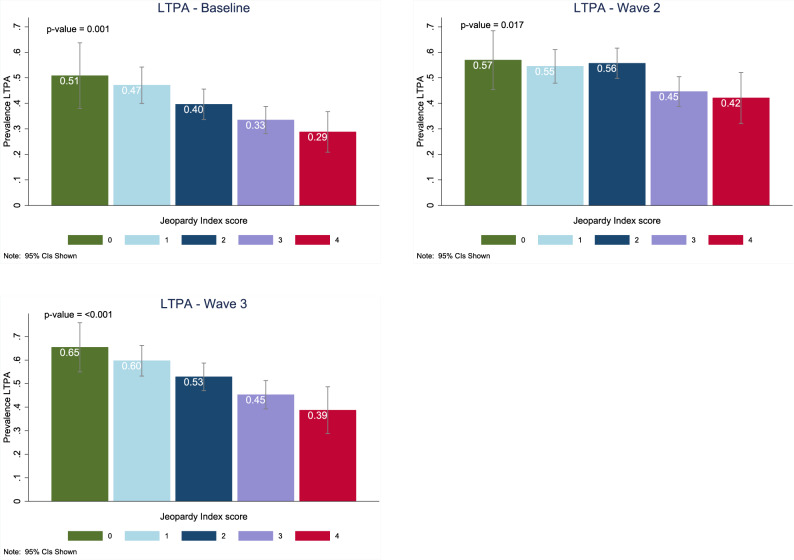
Prevalence of LTPA according to Jeopardy Index, across the three waves of the ISA study

The Jeopardy Index was inversely associated with LTPA prevalence across all waves (Fig. 2). Participants in the lower vulnerability group (score 0) consistently exhibited the highest LTPA levels, whereas those in groups with higher vulnerability (score 4) had the lowest prevalence. At baseline, LTPA prevalence was 51% in the least vulnerable group compared to 29% in the most vulnerable (*p* = 0.001). These inequalities slightly narrowed in wave 2, with a modest increase in LTPA observed across all groups, though the association remained significant (*p* = 0.017), possibly reflecting the COVID-19 pandemic context. In wave 3, disparities widened again, with the largest gap observed across index scores: LTPA reached 65% in the lower vulnerability group and 39% in the higher vulnerability group (*p* < 0.001).

**Fig. 3:**
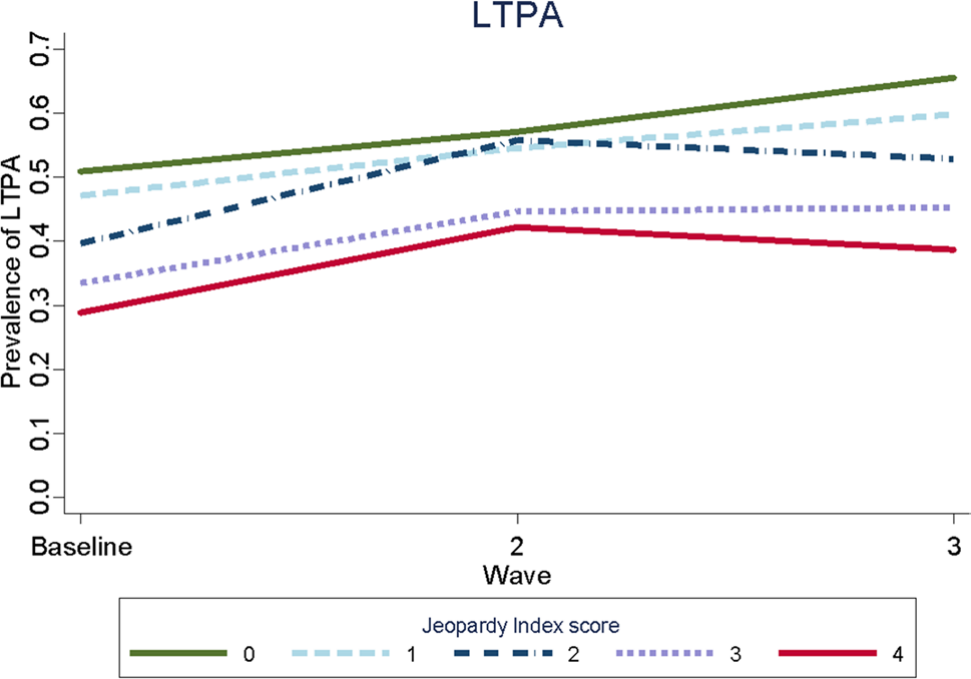
Prevalence of LTPA according to Jeopardy Index, along the three waves of the ISA study

Figure 3 illustrates the prevalence of LTPA along the three waves of the study, according to the Jeopardy Index score. Persistent disparities were observed throughout the period, with LTPA consistently higher among individuals with lower vulnerability (score 0) and lower among those with higher vulnerability (score 4). Over time, these inequalities became more pronounced: while the lowervulnerability group exhibited a continuous increase in LTPA, the higher vulnerability group showed a slight decline between waves 2 and 3. As a result, the gap between the lowest and highest Jeopardy scores widened, indicating that improvements in LTPA were unequally distributed.

**Fig. 4:**
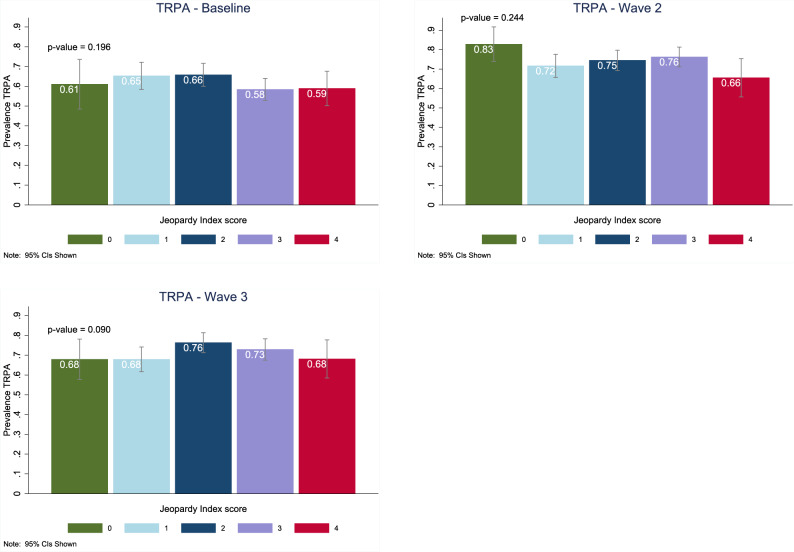
Prevalence of TRPA according to Jeopardy Index, across the three waves of the ISA study

Figure 4 presents the prevalence of TRPA according to Jeopardy Index scores, separately for each wave of the study. In all waves, there were no statistically significant differences in TRPA prevalence across social disadvantage groups (*p* > 0.05). Although some variations were observed, the lack of statistical significance suggests that TRPA was relatively stable across levels of cumulative social disadvantage over time.

**Fig. 5:**
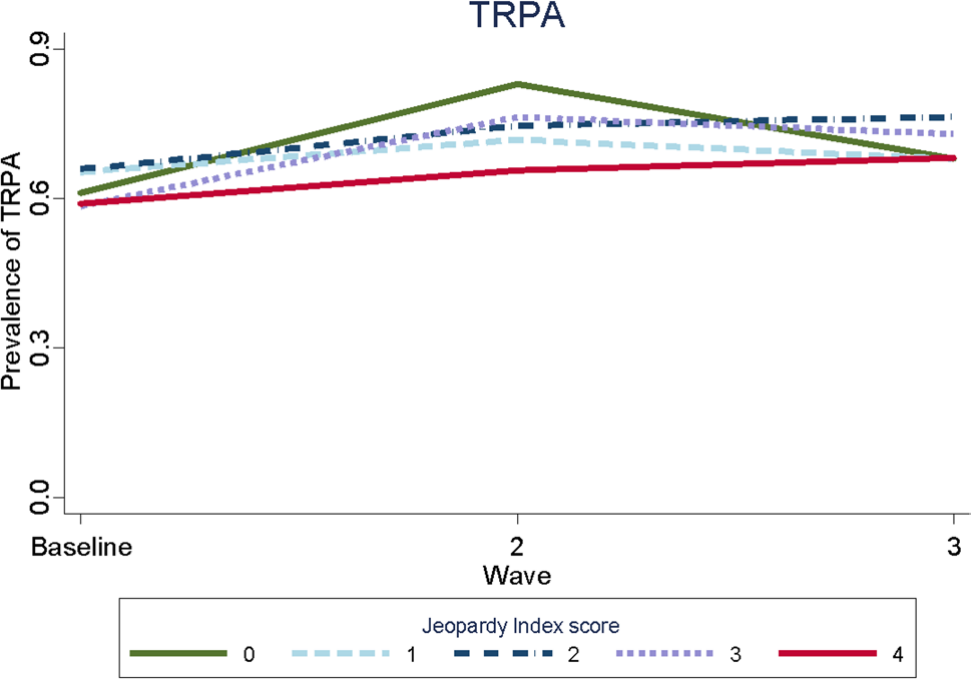
Prevalence of TRPA according to Jeopardy Index, along the three waves of the ISA study

Figure 5 shows the prevalence of TRPA across the three waves of the study, stratified by Jeopardy Index score. Overall, TRPA increased from baseline to wave 2 in all groups. Between waves 2 and 3, a slight decline was observed, particularly among individuals in the least vulnerable group (score 0), whereas TRPA levels continued to increase slightly in the most vulnerable group (score 4). Throughout the study period, individuals in the most vulnerable group consistently exhibited the lowest levels of TRPA. However, differences in TRPA across social vulnerability groups were less pronounced than those observed for LTPA.

An overview of the descriptive distributions of LTPA and TRPA across Jeopardy Index categories and according to covariates over the 10-year follow-up is provided in Supplementary Tables S2 and S3.

Table 5 then presents the association between the Jeopardy Index and both physical activity domains (LTPA and TRPA) over the same period (from baseline to wave 3).

**Table 5:** Association between Cumulative Social Disadvantage (Jeopardy Index) and Physical Activity Domains (LTPA and TRPA) over 10 Years of Follow-Up – ISA Study

	Jeopardy Index Score					*p* for trend
	0	1	2	3	4	
LTPA PR (CI 95%)						
Crude	Ref.	0.92 (0.76–1.12)	**0.85 (0.73–0.99)**	**0.70 (0.56–0.87)**	**0.61 (0.50–0.75)**	**< 0.001**
Adjusted	Ref.	0.93 (0.77–1.13)	0.86 (0.73–1.01)	0.72 (0.58–0.88)	**0.63 (0.52–0.77)**	**< 0.001**
Fully-adjusted	Ref.	0.94 (0.78–1.14)	0.86 (0.73–1.03)	**0.72 (0.56–0.91)**	0.64 (0.49–0.83)	**< 0.001**
TRPA PR (CI 95%)						
Crude	Ref.	0.96 (0.87–1.07)	1.01 (0.93–1.12)	0.97 (0.86–1.08)	0.89 (0.78–1.03)	0.258
Adjusted	Ref.	0.96 (0.87–1.07)	1.02 (0.93–1.12)	0.98 (0.88–1.09)	0.91 (0.79–1.05)	0.401
Fully-adjusted	Ref.	0.96 (0.87–1.05)	1.03 (0.94–1.13)	1.03 (0.92–1.15)	0.98 (0.84–1.15)	0.482

*Legend:* *PR* Prevalence Ratios, *95% CI* 95% Confidence interval, *Ref.* reference category

Crude: unadjusted model

Adjusted: adjusted for time (study wave)

Fully-adjusted: additionally adjusted for age group, marital status, employment status, BMI, and TV watching

*p for trend* was obtained by modeling the Jeopardy Index as an ordinal variable entered as a single continuous term, while categorical estimates are presented for interpretability

*Bold values indicate statistical significance (p < 0.05).*

A clear inverse gradient was observed, with decreasing prevalence of LTPA across increasing levels of cumulative social disadvantage. In the fully adjusted multilevel Poisson regression model, participants in Jeopardy categories 3 and 4, representing the most socially vulnerable groups, had significantly lower prevalence of LTPA compared to the reference group (category 0: male, White, and highly educated). Specifically, the prevalence ratio (PR) was 0.72 (95% CI: 0.57–0.91; *p* = 0.006) for category 3 and 0.62 (95% CI: 0.49–0.80; *p* < 0.001) for category 4. The test for trend was statistically significant (*p* < 0.001), indicating a dose–response relationship. In contrast, no significant association was found between the Jeopardy Index and TRPA in any of the models.

Sensitivity analyses using alternative cutoffs to define physical activity yielded results consistent with the main findings. When leisure-time physical activity was defined as meeting current physical activity recommendations (≥ 150 min/week), a clear inverse gradient across levels of cumulative social disadvantage persisted. In the fully adjusted multilevel Poisson model, participants in the highest Jeopardy categories showed substantially lower prevalence of meeting recommended levels of leisure-time physical activity compared with the reference group. Specifically, prevalence ratios were 0.61 (95% CI: 0.43–0.87) for category 3 and 0.55 (95% CI: 0.37–0.83) for category 4, with a statistically significant test for trend (*p* < 0.001). In contrast, no statistically significant associations were observed between the Jeopardy Index and transport-related physical activity when applying the ≥ 150 min/week cutoff.

## Discussion

This longitudinal study examined how intersecting social identities influence engagement in leisure-time and transport-related physical activity among Brazilians over a 10-year period. The findings revealed persistent and widening social inequalities in physical activity, particularly in the leisure-time domain. Despite overall increases in LTPA over time, individuals with lower vulnerability, particularly White men with higher educational attainment, remained consistently more active during leisure time. In contrast, individuals experiencing higher vulnerability, such as women from racialized or minority groups with lower educational attainment, exhibited significantly lower levels of LTPA, highlighting the compounding effects of social disadvantage. Notably, these disparities were not observed in the transport-related domain, suggesting that structural and contextual factors may influence physical activity differently across domains. These contrasting patterns underscore the importance of understanding physical activity not solely as an individual choice, but as a behavior shaped by intersecting social and environmental determinants.

The findings of this study align with existing literature on health behavior inequities and further demonstrate that different domains of physical activity are unequally distributed across social structure. Previous studies conducted in Brazil have shown that men are generally more active than women, and that individuals identifying with racial or ethnic minority groups consistently report lower levels of LTPA compared to their White counterparts, even after adjusting for income and education [34–36]. These patterns reflect broader social processes, as LTPA often depends on access to private facilities, green and safe environments, flexible work arrangements, time, social norms, and supportive networks, factors more readily available to socially advantaged groups [37]. In this context, socioeconomic factors play a central role in shaping both the awareness of, and the means to engage in, LTPA. Higher income and education levels are associated not only with greater knowledge of the health benefits of LTPA but also with improved access to resources such as time, services, and facilities, enabling participation in leisure activities [37, 38]. National data reinforce this trend, as shown by the 2019 National Health Survey [6], in which 43.3% of adults with higher education reported engaging in LTPA, compared to only 18.6% among those with up to elementary education. These figures mirror the disparities identified in this study and underscore the broader social determinants of LTPA participation.

In contrast, TRPA, such as walking, may reflect structural limitations, particularly among individuals who cannot afford private transportation or who live in poorly connected neighborhoods [39]. Several studies have reported that TRPA tends to be more prevalent among lower-income groups, highlighting inequities in urban mobility and access to motorized transport [40]. While walking for transport offers health benefits, it is not always a matter of choice but often necessity-driven [7–9]. However, in this study, TRPA was not significantly associated with cumulative social disadvantage, and differences across social groups were minimal, suggesting that transport-related activity may be more uniformly distributed or influenced by contextual factors such as public transportation availability, urban density, proximity to workplaces, and economic necessity, which warrant further investigation.

To better capture the complexity of social vulnerability, this study adopted an intersectional and multidimensional approach. While previous research often treats social categories such as sex, race/skin color, and education in isolation, intersectionality theory posits that these dimensions do not operate independently but rather interact to create unique and compounding experiences of privilege or oppression [19]. Traditional analyses based on single variables may obscure overlapping disadvantages and underestimate social inequalities. The Jeopardy Index, combining sex, race/skin color, and education into a single score, was used to capture intersectionality and revealed clear social differences in LTPA.

Evidence from Howard et al. [41] and Mielke et al. [22] indicates that intersecting disadvantages such as low income, female gender, and minority racial or ethnic status intensify structural and social barriers to engaging in health-promoting behaviors, with non-White women with low education and income consistently less likely to engage in LTPA. These cumulative effects may reflect racialized and gendered systems of exclusion, including unequal access to safe and attractive leisure environments, historical underinvestment in recreational infrastructure in racially marginalized neighborhoods, and routine experiences of discrimination in public spaces. Additionally, socially disadvantaged individuals are more likely to face time constraints due to long working hours, precarious employment, and caregiving responsibilities, which further reduce opportunities for LTPA [12, 42].

Notably, similar disparities in physical activity have been observed in other countries of the Global South, where structural inequalities and urban exclusion critically shape opportunities for engagement. Studies from Colombia, Mexico, India, African countries, and Chile report consistent gaps in LTPA by sex, education, and socioeconomic status, particularly affecting women and lower-income groups, due to a range of barriers such as caregiving responsibilities, unsafe environments, and sociocultural norms [13–18]. These findings reinforce that such inequities extend beyond Brazil and reflect broader global challenges. Yet, while identifying cumulative disadvantage is crucial for understanding how physical activity inequalities persist, translating this knowledge into effective policy remains a significant challenge [19–22].

In Brazil, addressing these persistent inequalities requires public health efforts that move beyond individual behavior change and adopt an intersectoral, equity-focused agenda, tackling broader social determinants through coordinated actions across urban planning, education, transportation, and public safety. Without such integration, policies risk perpetuating, rather than reducing, social inequities, as observed in other Global South contexts [18, 43–45].

Findings from this study underscore that LTPA remains largely a privilege of socially advantaged groups. Promoting inclusive physical activity requires not only investment in infrastructure but also addressing structural barriers such as income inequality, gender norms, and racial discrimination. Policies should follow the principle of proportionate universalism, tailoring interventions to levels of social vulnerability while ensuring equitable access to safe public spaces, affordable recreational opportunities, and inclusive mobility systems [1, 9, 46, 47]. Aligning these efforts with the UN Sustainable Development Goals can strengthen accountability and long-term commitments to reducing health disparities [27].

### Limitations and Strengths

While these findings offer important contributions to understanding the structural roots of health inequities, they should be interpreted in light of the study’s methodological limitations and strengths. First, the use of self-reported measures of physical activity may have introduced recall and social desirability bias, potentially leading to overestimation of activity levels, particularly among more advantaged groups [48]. Second, although the sociodemographic indicators employed offer valuable insights, they may not fully capture the complexity of social intersections that influence LTPA and TRPA. Nevertheless, the inclusion of sex, racial/ethnic identity, and education, provides a meaningful indicator of the hierarchical social structures that characterize Brazilian society. Another limitation concerns the generalization of the findings, as the sample was drawn from the urban population of São Paulo megacity and may not reflect the realities of other regions of Brazil. Additionally, common to longitudinal research is the attrition over time. However, multiple comparative analyses were conducted using data from all participants across the three follow-up periods, along with weighted analyses to match the baseline distribution of sex, age, and education. Findings from the sample of participants who completed all three waves did not differ meaningfully from the primary analyses, both in terms of sociodemographic characteristics and main outcomes.

Despite these limitations, the study presents important strengths. It is among the first in Brazil to investigate whether sex, racial/ethnic identity, and socioeconomic status intersect to influence physical activity during leisure and for transportation, using longitudinal data over a 10-year period from a large urban population in the Global South. By following the same cohort of individuals across three waves, we were able to capture the persistence, and in some cases, the widening, of social inequalities in physical activity. The longitudinal design enhances the ability to infer causal relationships and identify structural patterns that may be obscured in cross-sectional analyses. Furthermore, disaggregating physical activity into leisure-time and transport-related domains allowed for a more nuanced understanding of how each domain reflects distinct forms of social privilege or constraint. The integration of an intersectional lens, operationalized through the Jeopardy Index, enhances the study’s ability to account for multiple, overlapping axes of inequality. This approach not only improves the granularity of inequality measurement, but also aligns with recent calls in public health research to adopt more nuanced and equity-centered methodologies [49].

## Conclusions

By adopting a multidimensional and intersectional approach, this study underscores how cumulative social disadvantage shapes physical activity behaviors over time. Persistent and widening inequalities in LTPA were observed among Brazilians over a 10-year period, disproportionately affecting the most vulnerable groups, particularly women from racialized or minority groups with lower educational attainment. In contrast, no significant associations were found between cumulative disadvantage and TRPA, suggesting a more uniform distribution across social strata, potentially reflecting constrained choices rather than intentional, health-promoting behavior.

To effectively address these disparities, public policies and programs must account for the intersecting effects of race, sex, and education. Equity-focused and intersectoral strategies, integrating health promotion, education, urban planning, and social protection, are essential to create enabling environments that support physical activity for all, especially the most disadvantaged. Future research and policy development should be guided by intersectional frameworks to design inclusive interventions that tackle the structural roots of social inequalities in physical activity.

## Electronic Supplementary Material

Below is the link to the electronic supplementary material.

Supplementary Material 1

## Data Availability

Due to ethical considerations, the datasets generated and analyzed during this study are not publicly accessible. However, they can be made available upon reasonable request and subject to institutional approval. Data from the ISA – Physical Activity and Environment cohort study, along with related documentation, can be freely requested by completing the form available on the project’s official websites: www.isapacohort.net.br (English) or www.isaafcoorte.net.br (Portuguese). For further information, please contact: contact@isapacohort.br (English) or contato@isaafcoorte.br (Portuguese).

## Abbreviations

LTPA: Leisure-Time Physical Activity
TRPA: Transport-Related Physical Activity
WHO: World Health Organization
PAHO: Pan American Health Organization
IBGE: Brazilian Institute of Geography and Statistics
IPAQ: International Physical Activity Questionnaire
BMI: Body Mass Index
PR: Prevalence Ratios
95% CI: 95% confidence interval
ISA study: Health Survey of São Paulo: Physical Activity and Environment
