# Supplementary material for: Social inequalities in leisure-time and transport-related physical activity through the lens of intersectionality: 10-year longitudinal study in Brazil
Source: Int J Behav Nutr Phys Act. 2026 May 5;23:64. doi: 10.1186/s12966-026-01900-5 (PMC13312550; doi:10.1186/s12966-026-01900-5)
Supplement: Supplementary file 1 — Supplementary Material 1 [file 12966_2026_1900_MOESM1_ESM.docx]

**Supplementary Materials**

**Supplementary Figure S1.** Conceptual model supporting statistical analyses of the relationship between social inequalities and leisure-time and transport-related physical activity – ISA Study.


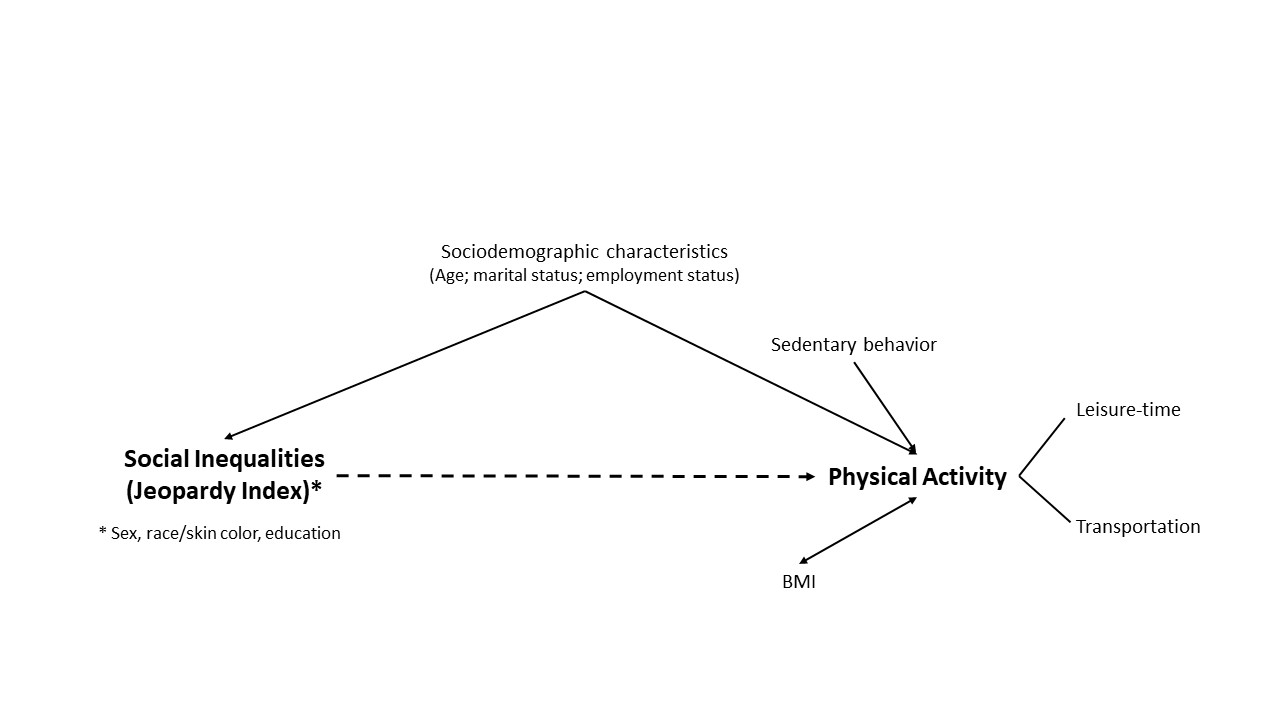


**Supplementary Table S1.** Pairwise associations between covariates and the Jeopardy Index assessed using Chi-square tests and Cramér’s V.

| Variable 1 | Variable 2 | Chi² | p-value | Cramér’s V | Strength of association |
| --- | --- | --- | --- | --- | --- |
| Age | **Marital status** | 411.85 | 0.000 | 0.377 | Moderate |
| Age | **Employment status** | 383.20 | 0.000 | 0.362 | Moderate |
| Age | **BMI** | 160.56 | 0.000 | 0.169 | Weak |
| Age | **Watching TV** | 54.47 | 0.000 | 0.136 | Weak |
| Age | **Jeopardy index** | 160.44 | 0.000 | 0.169 | Weak |
| Marital status | **Employment status** | 2.15 | 0.142 | 0.027 | Very weak |
| Marital status | **BMI** | 3.75 | 0.154 | 0.037 | Very weak |
| Marital status | **Watching TV** | 0.67 | 0.415 | 0.015 | Very weak |
| Marital status | **Jeopardy index** | 14.98 | 0.005 | 0.073 | Very weak |
| Employment status | **BMI** | 32.67 | 0.000 | 0.108 | Weak |
| Employment status | **Watching TV** | 79.94 | 0.000 | 0.165 | Weak |
| Employment status | **Jeopardy index** | 209.41 | 0.000 | 0.273 | Weak to moderate |
| BMI | **Watching TV** | 26.17 | 0.000 | 0.097 | Weak |
| BMI | **Jeopardy index** | 43.70 | 0.000 | 0.090 | Very weak |
| Watching TV | **Jeopardy index** | 18.49 | 0.001 | 0.081 | Very weak |

**Note:** Collinearity between covariates was assessed using the Variance Inflation Factor (VIF) in a linear model including all covariates. All VIF values were **below 5** (mean VIF = 1.43), indicating no collinearity concerns.

**Supplementary Table S2.** Prevalence of leisure-time and transport-related physical activity according to Jeopardy Index categories over 10 years of follow-up (ISA Study).

| Jeopardy Index | LTPA (%)* | TRPA (%)* |
| --- | --- | --- |
| 0 | 58.49 | 70.95 |
| 1 | 54.11 | 68.44 |
| 2 | 49.57 | 72.32 |
| 3 | 40.80 | 68.74 |
| 4 | 35.78 | 63.61 |
| *p for trend* | <0.001 | 0.130 |

* Percentages are based on pooled person-wave observations across all study waves during the 10-year follow-up. P-values for trend refer to Pearson’s chi-square tests.

**Supplementary Table S3.** Descriptive distribution of leisure-time and transport-related physical activity according to covariates over 10 years of follow-up (ISA Study).

| Covariate | LTPA (%) | p-value | TRPA (%) | p-value* |
| --- | --- | --- | --- | --- |
| Age group |  |  |  |  |
| 12 – 29 | 57.10 |  | 72.92 |  |
| 30 – 59 | 43.82 | <0.001 | 69.97 | <0.001 |
| 60+ | 43.34 |  | 64.49 |  |
| Marital status |  |  |  |  |
| with a partner | 43.78 | 0.001 | 66.62 | 0.003 |
| without a partner | 50.15 |  | 71.81 |  |
| Employment status |  |  |  |  |
| employed | 45.41 | 0.250 | 64.96 | <0.001 |
| unemployed | 47.55 |  | 71.74 |  |
| BMI |  |  |  |  |
| Without overweight | 49.19 |  | 72.78 |  |
| Overweight | 48.89 | <0.001 | 68.48 | <0.001 |
| obesity | 38.75 |  | 64.67 |  |
| Sedentary behavior (watching TV) |  |  |  |  |
| < 2 hours/day | 49.41 | 0.005 | 71.58 | 0.003 |
| > 2 hours/day | 44.25 |  | 66.47 |  |

* Values are row percentages. P-values were obtained using the chi-square test.
